# Association Between Workplace Bullying Occurrence and Trauma Symptoms Among Healthcare Professionals in Cyprus

**DOI:** 10.3389/fpsyg.2020.575623

**Published:** 2020-11-12

**Authors:** Loukia Aristidou, Meropi Mpouzika, Elizabeth D. E. Papathanassoglou, Nicos Middleton, Maria N. K. Karanikola

**Affiliations:** ^1^Department of Nursing, Faculty of Health Sciences, Cyprus University of Technology, Limassol, Cyprus; ^2^Mediterranean Hospital, Limassol, Cyprus; ^3^Faculty of Nursing, University of Alberta, Edmonton, AB, Canada

**Keywords:** PTSD, trauma, mental health, ICU, emergency, Cyprus, bullying, workplace incivility

## Abstract

Workplace bullying/mobbing is an extreme work-related stressor, but also a severe hazard for physical, mental and psychological health in healthcare employees, including nurses. A range of trauma-related symptoms has been linked with bullying victimization. The aim of the study was the investigation of workplace bullying/mobbing-related trauma symptoms in Greek-Cypriot nurses working in emergency and critical care settings, as well as of potential correlations with demographic and occupational variables. A descriptive, cross-sectional correlational study was performed in a convenience sample of 113 nurses. A modified version of the Part B.CII of The Workplace Violence in the Health Sector-Country Case Studies Research Instrument (WVHS-CCSRI Part C.II-M) and the modified Secondary Traumatic Stress Scale (STSS-M) were used for the assessment of bullying/mobbing frequency and workplace bullying/mobbing-related trauma symptoms, respectively. A total of 46.9% of the sample reported experiences of both bullying/mobbing victimization and witnessing of bullying/mobbing to others (VWB subgroup), 21.2% reported solely bullying/mobbing victimization (SVB subgroup) and 10.6% reported witnessing of bullying/mobbing to others (SWB subgroup). A total of 22.3% did not experience or witness any bullying/mobbing at the workplace. Trauma symptoms intensity (STSS-M total score) was more severe in the participants a) with a high frequency of workplace bullying/mobbing experiences compared to those with a moderate frequency of such experiences (*p* = 0.018), b) of the VWB subgroup compared to those of the SWB subgroup (*p* = 0.019), c) employed in Emergency Departments compared to those employed in ICUs (*p* = 0.03), d) who had considered resigning due to bullying/mobbing experiences compared to those who had never considered resigning (*p* = 0.008), e) who had been punished for reporting a bullying/mobbing incident compared to those who had not (*p* = 0.001), and f) who considered the incident unimportant to be reported compared to those who avoided reporting due to other causes (*p* = 0.048). This data highlights the need to establish effective and safe procedures for bullying/mobbing reporting, aiming to support bulling/mobbing victims and witnesses, and further to protect their legal rights. Both victims and witnesses of workplace bullying/mobbing need to be assessed by mental health professionals for PTSD symptoms in order to have access to effective treatment.

## Introduction

Bullying victimization and post-traumatic stress disorder (PTSD) symptoms have been both identified as occupational hazards for healthcare professionals, including nurses (Lanschinger and Nosko, 2015). Bullying victimization refers to any kind of unethical behavior (physical, verbal, and interpersonal), which is systematically expressed aiming to humiliate and disempower the “target-victim” (Becher and Visovsky, 2012). Accordingly, workplace bullying regards actions of humiliation, isolation or removal of an employee from a workplace, project or work-related situation (Carter et al., 2013). Bullying may be expressed by an individual or a group of people toward the target employee (Carter et al., 2013), while there is a power imbalance between the victim and the perpetrator(s) (Leymann, 1996). Mobbing is a term used interchangeably to bullying (Branch et al., 2013). However, according to some scholars, “bullying” regards victimization of the target employee by one perpetrator, while “mobbing” is used when there is a group of perpetrators (European Agency for Safety and Health at Work, 2010; Professional Issues Panel on Incivility, 2015).

Post-traumatic stress disorder symptoms are experienced after exposure to a severe traumatic event (American Psychiatric Association, 2013). These symptoms constitute a syndrome, encompassing intense arousal and agitation, re-experiencing of the traumatic event and avoidance of stimuli linked to the traumatic event. Persistent cognitive and mood disturbances, as well as enduring dysfunction in one’s personal and social life are also prominent in this syndrome (American Psychiatric Association, 2013).

Although PTSD and response to bullying victimization share similar manifestations, e.g. distressing emotions, functional impairment and physiological arousal (Tatar and Yüksel, 2019), there is limited data on the association between these phenomena in nurses employed in highly stressful work environments, such as emergency departments (EDs) or intensive care units (ICUs) (Mealer et al., 2012). Previous studies have identified the link between PTSD symptoms and exposure to workplace bullying, however in different populations, i.e. physicians (Tatar and Yüksel, 2019).

Differences between nurses and physicians regarding bullying/mobbing prevalence, as well as variations of the bullying/mobbing frequency in nursing population across studies warrant further research. Nevertheless, according to international literature, workplace bullying/mobbing is frequent among healthcare professionals (Zapf and Einarsen, 2011; Norton et al., 2017). Specifically, bullying/mobbing incidence ranges between 14.2 and 53.1% in physicians (Chatziioannidis et al., 2018; Cheung et al., 2018), while this frequency has been reported between 2.4 and 81% in nurses (Bambi et al., 2018). Other data reports that almost half of the healthcare personnel employed in emergency care services report bullying/mobbing victimization (Fullerton et al., 2019). Moreover, in contrast to international literature, it seems that nurses in Cyprus experience more frequently bullying/mobbing victimization than physicians. Specifically, in a study among healthcare professionals in Cyprus, nurses reported more frequently exposure to a bullying/mobbing incident than physicians (53.3 vs 31.4%, respectively) (Zachariadou et al., 2018), while in a systematic review the prevalence of workplace bullying/mobbing toward nurses and physicians was almost the same (30.8 and 31.9%, respectively) (Lever et al., 2019).

Although bullying/mobbing in nurses seems to be present in all work settings there are work environments in which employees are more often exposed to it (Bambi et al., 2019). Previous data reports a bullying/mobbing frequency of 25.2 to 59.3% in operating rooms (Park et al., 2014; Halim and Riding, 2018), 29 to 53.5% in critical care settings (Yun et al., 2014; Ganz et al., 2015; Chatziioannidis et al., 2018), as well as up to 90% in EDs (Al-Ghabeesh and Qattom, 2019).

According to some researchers, bullying may be deemed as a learned behavior, highly dependent on the culture prevailing in a work environment (Vessey et al., 2011), which supports the evidence on differences regarding the prevalence of workplace bullying in various work settings. However, it is difficult to compare any data on bullying prevalence since relevant studies have applied different definitions, methodologies and tools to measure this phenomenon. Moreover, the work conditions and inherent stressors vary across work settings. Overall, although the link of work-related stress with exposure to workplace bullying/mobbing and PTSD symptoms has been partially explored in healthcare professionals, relevant data is controversial regarding emergency and critical care nurses, warranting further investigation (Kim et al., 2019). In any case, EDs and ICUs are considered as highly stressful work environments compared to other work settings (Efe and Ayaz, 2010). Indeed, previous data confirms increased work-related stress and PTSD symptoms in emergency and critical care nurses (Karanikola et al., 2015). For instance, a study on the prevalence of bullying in Jordanian ED nurses revealed than almost 90% of the sample reported bullying/mobbing victimization (Al-Ghabeesh and Qattom, 2019).

Furthermore, little is known about the association of bullying/mobbing incidence and relevant trauma symptoms with adverse work-related phenomena (e.g. job dissatisfaction, professional burnout, poor communication among colleagues, etc.), or socio-demographic, educational and occupational characteristics in emergency and critical care nurses (Kendall-Gallagher et al., 2017; Vahedian-Azimi et al., 2019). Previous studies in ED nurses have shown that the majority of those who were bullied reported decreased productivity and declined ability to respond to cognitive demands, as well as to provide support, effective communication and safe care (Al-Ghabeesh and Qattom, 2019). Overall, a number of job-related problems have been linked with bullying/mobbing exposure in nurses (Olsen et al., 2017; Finstad et al., 2019), while there is considerable evidence on the positive association between bullying/mobbing victimization and burnout in them (Waschgler et al., 2013; Allen et al., 2015; Kim et al., 2019).

Apart from burnout, exposure to workplace bullying/mobbing has been linked with physical problems (Vignoli et al., 2015; Sauer and McCoy, 2017; Choi et al., 2018), while there is a gap in the evidence regarding the association of bullying victimization with severe psychological and mental health problems in healthcare professionals, mainly those employed in emergency and critical care settings. Previous studies in hospital nurses have shown the association of workplace bullying/mobbing experiences with low self-esteem, sadness, depressive and anxiety symptoms, as well as substance use and suicidality, a very severe mentally distressing condition (McKenna et al., 2003; Dumont et al., 2012). Specifically, according to a systematic review, suicidal ideation has been described in healthcare professionals who had been bullying/mobbing victimized (Leach et al., 2017). Increased frequency of suicide attempts has been reported in medical students, targets of bullying/mobbing (Frank et al., 2006), while suicidal ideation has also been associated with workplace bullying victimization in paramedics (Sterud et al., 2008). Yet, relevant data on emergency and critical care nurses is still scarce. Additionally, other studies have shown the relation between exposure to bullying/mobbing and personal life distress (Oriol et al., 2019). The Word Health Organization persistently highlights work-related risks as a public health issue, with special focus on the healthcare workforce (World Health Organization [WHO], 2010). Work-related bullying and PTSD in healthcare professionals may lead to a significant burden for both individuals and society, while psychological, physiological and mental distress in healthcare workers is associated with the safety and quality of patient care (Hodgins et al., 2014; Chatziioannidis et al., 2018).

The present study, for the first time, explored the link between workplace bullying/mobbing victimization and relevant trauma symptoms in critical and emergency care nurses in Cyprus. Evidence on the association between workplace bullying/mobbing and mental health sequelae can inform empowerment programs for healthcare employees who experience distress due to exposure to workplace bullying/mobbing. Specifically, data on the socio-demographic and employment profile of those who experience more frequently bullying/mobbing-related trauma symptoms can help identify groups at risk, and inform targeted implementation of preventive and interventional programs.

## Aim

The purpose of this study was to investigate workplace bullying-related trauma symptoms in Greek-Cypriot nurses working in emergency and critical care settings. Specifically, it was hypothesized that these symptoms are associated with a) socio-demographic, educational, and occupational variables, and b) indicators of work-related stress, i.e. emotional exhaustion, low work satisfaction, low satisfaction from relations with colleagues, as well as indicators of personal distress, i.e. low satisfaction from personal life and social relationships. Also, it was hypothesized that factors (a) and (b) may be predictors of workplace bullying-related trauma symptoms. An additional objective was to explore any association between workplace bullying/mobbing experience and a) socio-demographic, educational, and occupational variables, and b) indicators of work-related stress, i.e. emotional exhaustion, low work satisfaction, low satisfaction from relations with colleagues, as well as indicators of personal distress, i.e. low satisfaction from personal life and social relationships. Thus, an association between bullying/mobbing experience and variables (a) and (b) was also hypothesized.

## Materials and Methods

### Design

A descriptive, correlational design with cross-sectional comparisons was applied.

### Sampling

The target population was emergency and intensive care nurses working in private and public healthcare services in Cyprus. Given the lack of previous evidence on the associations pursued, the sample size was calculated through power analysis to be 98 nurses [α = 0.05 statistical significance level, moderate correlation effect (0.3–0.4) with 80% statistical power]. Inclusion criteria were: a) employment in ED, Coronary Critical Care Unit (CCCU) or ICU for more than 12 months, a criterion relevant to the assessment tool for the bullying/mobbing phenomenon, i.e. “In the last 12 months, have you been bullied/mobbed in your workplace?” (World Health Organization [WHO], 2003); b) comprehension of the Greek language; and c) written informed consent.

### Study Environment

The four public adult hospitals based in Paphos, Larnaca, Limassol, and Nicosia were invited to participate in the study, as well as all five private ones. Regarding the public sector, there is one ICU, one CCCU and one ED in the hospitals of Nicosia and Limassol, and one ED and a general ICU in the hospitals of Paphos and Larnaca. The study environment was the four ICUs (roughly 12 beds per unit), the four EDs (providing services to nearly 80 individuals per shift) and the CCCU of Limassol (approximately 10 beds per unit). The CCCU based in the Nicosia public adult hospital although invited, did not agree to participate in the study. Eventually, four ICUs, four EDs and one CCCU from the public sector participated in the study. One of the five private hospitals in the Republic of Cyprus agreed to participate in the study. This hospital is based in Limassol and includes one CCCU (11 beds) and one ICU (7 beds), while the ED provides services to almost 50 individuals per shift.

### Data Collection

Collection of data took place from October 2018 to February 2019 via a questionnaire that included three sections, as follows: socio-demographic, educational and employment data section, tools section and a separate section for the printed consent form. The principal investigator (AL) distributed 347 questionnaire packages to the study units. The completion time for each questionnaire package was 8–10 min.

The following variables were included in the socio-demographic, educational and employment data section: age group, gender, marital status, education level, type of work setting (ICU, CCCU, and ED), type of work hospital (private/public), work province, work experience, employment status (shifts/no shift), immigrant/minority group. Additionally, assessment of the degree of emotional exhaustion, work satisfaction, satisfaction from relations with colleagues, satisfaction from personal life and satisfaction from social relationships through Visual Analog Scales (VAS), ranging from 1 to 10 (higher values indicating increased satisfaction), was included. The reason for assessing these variables was that emotional exhaustion and work satisfaction are features associated with prolonged exposure to a stressful work environment, and so is the intention to quit the job (Schadenhofer et al., 2018). Satisfaction from relations with colleagues may also be associated with workplace bullying/mobbing phenomena (Bambi et al., 2018), while satisfaction from personal and social life has been associated with experience of PTSD symptoms (American Psychiatric Association, 2013).

The tools section included the Greek version of Part C.II (Mobbing/Bullying) of the Workplace Violence in the Health Sector-Country Case Studies Research Instruments (WVHS-CCSRI), previously translated, modified and validated by Zigrika et al. (2013), hereinafter referred to as WVHS-CCSRI Part C.II-M. The WVHS-CCSRI Part C.II-M was used herein for the assessment of workplace bullying/mobbing variables (World Health Organization [WHO], 2003; Zigrika et al., 2013), as it is the only part of the WVHS-CCSRI, that aims to provide descriptive information about workplace bullying/mobbing. The original Part C.II of the WVHS-CCSRI includes 14 items in a combination of categorical variables and Yes/No response variables. Different forms of violence, e.g. sexual harassment, addressed by the WVHS-CCSRI were beyond the scope of this investigation, thus not included herein. Also, Part C.II of the WVHS-CCSRI has been used as a stand-alone instrument before (Zigrika et al., 2013).

The modifications included in the WVHS-CCSRI Part C.II-M are listed below (Zigrika et al., 2013). Specifically, aiming to acquire additional information on the bullying/mobbing phenomenon, seven items were added according to relevant literature. Also, several items were transformed and one item was eliminated from the original Part C.II (see below). Thus, the final number of items included in the WVHS-CCSRI Part C.II-M was 19. One of the added items addresses witnessing bullying/mobbing to others, based on evidence that just witnessing this phenomenon may have a negative impact on one’s health status (Lutgen-Sandvik, 2006; Branch et al., 2013): “In the last 12 months have you witnessed bullying/mobbing toward other people in your workplace? (Yes/No).” Aiming to assess the degree of power imbalance between the victim(s) and the perpetrator(s) (Leymann, 1996), the following item was added: “The last time you were bullied/mobbed, the person who intimidated you a) was of equal ranking, b) was of superior ranking, c) was of inferior ranking, d) had no ranking relation with you.” The item “How was the bullying/mobbing expressed (Verbally; Physical violence/gestures; Gossip/rumors; Isolation/Information hiding; Irrelevant duties or assignments/unrealistic deadlines assignment)?” was added to assess the different forms of workplace bullying/mobbing (Branch et al., 2013), while the item “Have you ever been punished because you reported a workplace bullying/mobbing incident? (Yes/No)” was added to assess the factors associated with one’s intention to report or not a bullying/mobbing incident (Chen et al., 2009; Mantzouranis et al., 2015). The items “How satisfied are you with the way you handled the most recent bullying/mobbing behavior you have experienced in your workplace? (Please indicate your response from 1 to 10)” and “How did you respond to the most recent incident of workplace bullying/mobbing that you witnessed? (Please tick all relevant boxes: I advised the bullying victim to kindly ask the bully to stop this behavior; I myself reported the workplace incident to the manager; I myself asked the bully to stop; I advised the bullying victim not to take action until the incidence was repeated; I took no action)” were added to assess the link between one’s response and the degree of experienced distress (Mantzouranis et al., 2015; Berry et al., 2016). The item “Have you ever resigned or considered resigning because of bullying/mobbing experiences in your workplace? (Yes, I did; I thought of it but did not; No)” was added to assess the impact of the phenomenon on employees’ morale, since associated phenomena such as moral distress have been linked with one’s intention to quit job (Vessey et al., 2009; Bambi et al., 2018). The item “How often, in your opinion, bullying/mobbing happens in your workplace? (no risk; low; moderate; high risk)” was added to assess the participants’ perception about the safety in their workplace (Blando et al., 2013).

The responses about the frequency of bullying/mobbing as included in the BM2 item of the original Part C.II of the WVHS-CCSRI were transformed as follows: “Almost every day” instead of “all the time,” “4–5 times per year” instead of “sometimes” and “1–2 times in total” instead of “once.” The aim was to get more accurate data on the frequency of the responses. Similarly, the item BM4 of the original questionnaire was transformed as follows: “How often, in your opinion, bullying/mobbing happens in your workplace? (Rarely/never; Not very frequently; Moderately frequently; Very frequently; Highly frequently).” The responses on the items BM3 and BM5 of the original questionnaire were also transformed to reflect the ICU/CCCU/ED environment. An open question about the description of possible ways to prevent relevant phenomena was added to the original item BM8. Similarly, the original BM 9.1 item was transformed to an open question asking the responder to name the person who investigated the bullying/mobbing incident. This open question was incorporated to the item “Was any action taken to investigate the causes of the bullying/mobbing?” The response “no support at all” was added to the original BM10 item in order to get additional information. Similarly, two responses were added to the original item BM6, as follows: “Tried to defend myself physically,” “Asked support from the anti-bullying committee of the hospital.” Two more responses were added to the original BM12 item as follows: “Anti-bullying policy is not provided by the institution” and “Colleagues and peers asked/advised me not to report/take actions against.” Moreover, the original BM 7 item on the impact of bullying/mobbing on the victim was eliminated, since a structured scale for the assessment of workplace bullying/mobbing related distress was further included in the present tools section. The WVHS-CCSRI Part C.II-M applied in the present study is included as a Supplementary Material.

A definition of the term “bullying/mobbing” as a recurring and over time malicious and offensive physical, verbal or interpersonal behavior aiming to humiliate and undermine an employee or a group of employees by a person or group of persons (Leymann, 1996) was included at the beginning of the WVHS-CCSRI Part C.II-M. This definition is in line with the glossary of the WVHS-CCSRI (World Health Organization [WHO], 2003), while at the same time integrates the twofold dimension of the phenomenon (personal or group dimension). It is clarified that both “”bullying” and “mobbing” are expressed by the same word in the Greek language, thus, it was necessary to include the description of both dimensions (personal and group dimension) of the phenomenon in the definition. Therefore, both terms, i.e. “Bullying” and “Mobbing,” have been used in this study.

A modified version of the Secondary Traumatic Stress Scale (STSS-M) (Bride et al., 2004) was used for the assessment of the frequency of symptoms of the traumatic response due to exposure to workplace bullying/mobbing. The original STSS was developed to assess secondary (vicarious) traumatic stress symptoms in healthcare professionals, rather than symptoms resulting from a direct exposure to a traumatic event. Nonetheless, we deemed it as an appropriate tool for the assessment of workplace bullying-related trauma symptoms, as STSS items were developed according to PTSD symptoms, as described in the Diagnostic and Statistical Manual for Psychiatric Disorders- IV-Text Revised, which are relevant to a direct exposure to a severely traumatic event (American Psychiatric Association, 2013). Furthermore, the statements of the items of the original STSS were properly modified to ensure that they were suitable to experiences of personal bullying/mobbing victimization or witnessing of bullying/mobbing victimization to others. Examples of the modified statements are “It seemed as if I was reliving the bullying/mobbing experience I had” or “I had trouble sleeping after the bullying/mobbing experience I had.” The modified STSS (STSS-M) included 17 items, as did the original instrument.

The 17 modified items included in the STSS-M were rated on a 5-point Likert scale, from 1 (never) to 5 (very often), similarly to the items included in the original tool. Specifically, the participants were asked to report the frequency at which they experienced the symptoms described in each statement during the previous 2 weeks. Similarly to the original, the total STSS-M score ranged from 17 to 85, with higher scores reflecting more intense self-reported workplace bullying/mobbing-related trauma symptoms. The 17 items of the STSS-M were grouped into intrusion, arousal and avoidance symptoms, reflecting the three subscales of the STSS-M. Cronbach’s alpha internal consistency reliability coefficient of the original STSS has been reported as 0.93 (Bride et al., 2004). The construct validity of the STSS has been also assessed by Bride et al. (2004) through confirmatory factor analysis, providing values showing adequate model fit (GFI = 0.90. CFI = 0.94, IFI = 0.94, and RMSEA = 0.069). Similarly to the original, the STSS-M may be used as a three-dimensional instrument, as well as an uni-dimensional tool (Bride et al., 2004). The STSS-M was used herein as an uni-dimensional tool. The internal consistency reliability via Cronbach’s alpha value was assessed for the STSS-M tool, as well as test-retest reliability with Pearson’s *r* score. Specifically, test-retest reliability was assessed with repeated administration of the tool within 1-week time interval in 20 responders. Only the responses of the first measurement were included in the final sample. Regarding the test-retest reliability, Pearson’s *r* score for the two assessments was 0.91(*p* < 0.001). Cronbach’s Alpha coefficient for the internal consistency reliability was 0.942.

### Ethical Issues

The participants were informed both orally and in written form about the purpose and process of the study, as well as about confidentiality issues regarding the revealed information and the safe storage of collected data. Specifically, the information and consent form explained the objectives, anticipated results and procedures of the study. Moreover, anonymity and the voluntary nature of participation in the study were all assured orally and in the consent form, while the participants were able to ask questions. They were also assured that there would be no repercussions in their work status if they did not participate in the study. The main researcher (LA) remained at each study setting for about an hour to answer any questions raised by the participants.

Each questionnaire package was provided in an open, non-transparent envelope, with no identifying characteristics. Questionnaires were anonymous. The participants were informed that they had to place their completed questionnaire in the envelope, to seal it and then to put in a box, located in their work place. The box was also non-transparent, while removal of envelopes was not possible. Only the principal investigator (LA) had access to the key of each box. It is clarified that there were no work or hierarchical relationships between the principal investigator, the research team and the participants.

Information on how to contact the research team and the relevant Ethics Board was included in the consent form, in case the participants had any questions about the questionnaire or the procedure. Permission to conduct the study was obtained from the National Committee of Bioethics of Cyprus [NCBC EP 2018.01.110] and the Research Promotion Committee of the Ministry of Health of Cyprus [MH 5.34.01.7.6E-0482/2018].

### Data Analysis

The participants who were victimized and/or witnessed workplace bullying/mobbing completed the STSS-M questionnaire, and associations were assessed with a) demographic, educational and employment variables, and b) bullying/mobbing descriptive variables. Frequencies were assessed for categorical variables and mean value (M) and standard deviation (SD) for continuous ones. The age and work experience were transformed from continuous into categorical variables, according to relevant literature (Bambi et al., 2018). Continuous variables were checked for normality and the parametric measures *t*-test and ANOVA were applied for comparisons among groups. For significant differences among multiple groups, post-hoc analyses were carried out with the Scheffe test. The chi square test was used for comparisons between groups regarding categorical variables. Pearson’s r was used to determine correlation between numerical variables. Aiming to model the predictors of workplace bullying-related trauma symptoms (STSS-M total score > 17) a multivariate analysis via stepwise logistic regression was applied. Specifically, the total STSS-M score was transformed into a categorical dichotomous variable (dependent variable) and the following were included as the independent variables: a) socio-demographic, educational, and occupational variables, and b) emotional exhaustion, work satisfaction, satisfaction from relations with colleagues, satisfaction from personal life and satisfaction from social relationships.

The level of statistical significance was set at <0.05. The statistical package IBM SPSS (version 25. 0) was used for data analysis.

## Results

### Socio-Demographic and Employment Characteristics

Of the 347 questionnaires distributed, 117 questionnaires were returned. Four of them were excluded as incomplete (valid response rate: 32.56%). The final sample included 113 nurses (42.4% males, 57.5% females). Participants’ mean age was 32.9(+7.6) years, most of them were Greek-Cypriots (94.7%), while 46.9% were single. Nearly 40.7% of the sample had a Master’s degree. Approximately 55.8% were employed in adult ICU and 29.2% in ED, while 29.2% were employed in the private sector. The mean overall clinical experience of participants was 6.85 years (±6.5), while their mean work experience in the current position was 2.26 years (±0.8). Approximately 92.9% of the sample were staff nurses (Table 1).

**TABLE 1:** Socio-demographic, employment and educational data of the sample (*n* = 113).

		*N*	%
Gender	Female	65	57.5
	Male	48	42.5
Marital status	Single	53	46.9
	Married	60	53.1
Education	Bachelor degree only	67	59.3
	Post-graduate diploma/Master’s degree	46	40.7
Employment sector	Private	33	29.2
	Public	80	70.8
Ward	ICU	63	55.8
	CCCU	17	15.0
	ED	33	29.2
Ranking	Head/Under head nurse	8	7.1
	Staff nurse	105	92.9
Did you move from abroad to be employed as a nurse in Cyprus?	Yes	6	5.3
	No	107	94.7
	**Mean value**	**Standard deviation**	
Age (years)	32.9	7.6	
Work experience in the current potion (years)	2.26	0.8	
Overall work experience in nursing (years)	6.85	6.5	

*ICU, intensive care unit; CCCU, coronary critical care unit; ED, emergency department.*

The mean were [M(SD)] for the reported emotional exhaustion, work satisfaction, satisfaction from relations with colleagues, satisfaction from personal life and satisfaction from social relationships were 6.5(2.2), 6.2(1.8), 7.2(1.7), 7.5(1.6), and 7.6(1.6) [Scale range (SR): 1–10], respectively. All these values indicate a moderate to high degree of these variables.

### Bullying/Mobbing Variables as Described by the WVHS-CCSRI Part C.II-M Questionnaire

Nearly 78.8% (*n* = 89) of the sample reported a bullying/mobbing relevant experience, i.e. bullying/mobbing victimization, bullying/mobbing witnessing to others, or both. Specifically, almost half of the participants (*n* = 53, 46.9%) reported both victimization and witnessing bullying/mobbing to others (VWB subgroup), while 21.2% (*n* = 24) reported that they had been solely bullying/mobbing victimized (SVB subgroup). Also, 10.6% (*n* = 12) of the sample reported witnessing bullying/mobbing to others, while they had not been victimized themselves (SWB subgroup). Those who reported that they had neither witnessed nor been victimized formed the “no bullying/mobbing experience” subgroup (NB). This group represented the 21.2% of the sample. In summary, the VWB and SVB subgroups included those who reported bullying/mobbing victimization, accounting for the 68.1% of the sample (*n* = 77), while the VWB and SWB subgroups represented those who reported witnessing workplace bullying/mobbing to other employees, accounting for the 57.5% of the sample (*n* = 65).

### Mean Differences Between Bullying/Mobbing Groups

A statistically significant difference was found between the bullying/mobbing subgroups (i.e. VWB, SWB, SVB, and NB) regarding the degree of emotional exhaustion (ANOVA, *F* = 3.640, *p* = 0.015). Specifically, the responders who reported no bullying/mobbing experiences reported lower degree of emotional exhaustion [M(SD): 5.70(2.19) – SR: 1–10] compared to those who reported experience of both bullying/mobbing victimization and witnessing of bullying/mobbing to others [M(SD): 7.20(2.09) – SR: 1–10] [95% CI: 0.58 (−0.035–3.033), *p* = 0.049)]. No other statistically significant differences were found among the different bullying/mobbing subgroups and a) the degree of work satisfaction, b) satisfaction from relations with colleagues, c) satisfaction from personal life, and d) satisfaction from social relationships. In relation to socio-demographic and employment characteristics, there was no statistically significant association between gender and bullying/mobbing subgroups. In contrast, those aged between 26 and 35 years reported more frequently (63.9%) bullying/mobbing victimization compared to those being younger than 25 years (5.2%) and older than 35 years (31.2%) (*x*^2^, *p* = 0.003). Also, the participants with work experience less than 5 years reported more frequently (46.8%) experience of witnessing bullying/mobbing to others than those with 6–10 years (32.1%) or more than 11 years (22.1%) of work experience (*p* = 0.003). Married participants reported less frequently (27.8%) an intention to quit the job due to workplace bullying/mobbing victimization than single ones (72.2%) (*p* = 0.003).

With regard to educational level only borderline significances were noted. The participants with a Master’s degree witnessed workplace bullying/mobbing to others more frequently (53.8%) than those with only a Bachelor’s degree (46.2%) (*p* = 0.001). Also, Master’s holders reported more frequently intention to quit the job due to bullying/mobbing victimization (61.1%), than those with a Bachelor’s degree only (38.9%) (*p* = 0.046).

With regard to employment sector, the participants employed in the public sector reported more frequently bullying/mobbing victimization and witnessing of bullying/mobbing to others (84.4 and 81.5%, respectively), than those employed in the private sector (15.6 and 18.5%, respectively) (*p* < 0.001, *p* = 0.003, respectively).

Statistically significant associations were noted between district of employment and bullying/mobbing variables, regarding bullying/mobbing victimization (*p* = 0.004), as well as witnessing of bullying/mobbing to others (*p* = 0.016). Regarding clinical unit, those employed in ICUs reported more frequently bullying/mobbing victimization (61.6%) compared to ED (29.3%) and CCCU (9.1%) participants (*p* = 0.031). Also, 61.1% of the ED responders thought of quitting the job due to bullying/mobbing victimization compared to 38.9% of the ICU and 0% of the CCCU responders (*p* = 0.015). Finally, in relation to the self-perceived risk for bullying/mobbing in the work department, 81% of the ICU responders and 9.5% of both the ED and CCCU responders reported a moderate risk of relevant phenomena (*p* = 0.012).

### Bullying/Mobbing-Related Trauma Symptoms as Described by the STSS-M Questionnaire

Approximately 93.3% of those reporting any bullying/mobbing experience (i.e. victimization, witnessing or both) also reported relevant trauma symptoms, according to the STSS-M score. Specifically, 57.3% reported rare manifestations of trauma symptoms, while 20.2%, 14.6%, and 1.1.% reported occasional, often and very often manifestations of bullying/mobbing-related trauma symptoms, respectively. Thus, 35.9% of the sample reported above moderate frequency of bullying/mobbing-related trauma symptoms.

Furthermore, the most frequent traumatic response was reported in relation to the item “I wanted to avoid working with some people linked with the bullying/mobbing experience I had (STSS-M item 14).” This item also reflected the most frequently reported symptom in the categories “often”/“very often” (*n* = 22, 24.7%), along with the item “I was easily annoyed after the bullying/mobbing experience I had (STSS-M item 15)” (*n* = 18, 20.22%).

Table 2 presents mean and standard deviation scores in each STSS-M item, as well as mean differences in STSS-M scores across the three subgroups of the participants as described by the WVHS-CCRI Part C.II-M questionnaire (SWB, SVB, VWB). Specifically, a statistically significant difference was noted between those who were VWB and those who only witnessed bullying/mobbing to others (SWB) regarding the frequency of the experienced trauma symptoms as expressed by the STSS-M score (Table 2). In particular, the VWB subgroup reported higher values in STSS-M compared to the SWB subgroup in the following items: “My heart started pounding when I thought about the bullying/mobbing experience I had (STSS-M item 2)” (*p* = 0.028), “I felt discouraged about the future after the bullying/mobbing experience I had (STSS-M item 5)” (*p* = 0.030), “I felt jumpy after the bullying/mobbing experience I had (STSS-M item 8)” (*p* = 0.048), and “I wanted to avoid working with some people linked with the bullying/mobbing experience I had (STSS-M item 14)” (*p* = 0.006). Additionally, the reported trauma symptoms were more frequent in the VWB subgroup compared to the SVB subgroup regarding the item “I was easily annoyed after the bullying/mobbing experience I had (STSS-M item 15)” (*p* = 0.027). Finally, the overall intensity of the workplace bullying-related trauma symptoms as assessed by the total STSS-M frequency score was higher in the VWB subgroup compared to the SWB subgroup (*p* = 0.019).

**TABLE 2:** Differences in the intensity of workplace bullying-related trauma symptoms between the participants who only witnessed bullying/mobbing (SWB), those who were bullying/mobbing victimized (SVB), and those who were both victims and witnesses of bullying/mobbing (VWB) in the workplace.

		95% Confidence Interval for Mean								
Workplace bullying-related trauma symptoms (STSS-M statements)	Sample subgroup	*N*	STSS-M item Mean	Std. Deviation	Std. Error	Lower bound	Upper bound	*P* (ANOVA)	*Post-hoc analysis* (Scheffe test)	
In the previous 2 weeks:									p	Subgroups: Std. Error (95% CI)

1. I felt emotionally numb after the bullying/mobbing experience I had.	Solely bullying/mobbing victims	24	2.42	1.316	0.269	1.86	2.97			
	Solely witnesses of bullying/mobbing to others	12	1.92	0.793	0.229	1.41	2.42			
	Both victims and witnesses pf bullying/mobbing	53	2.60	0.968	0.133	2.34	2.87			
								0.127		
2. My heart started pounding when I thought about bullying/mobbing experience I had.	Solely bullying/mobbing victims	24	2.17	1.167	.238	1.67	2.66			
	Solely witnesses of bullying/mobbing to others	12	1.33	0.492	0.142	1.02	1.65		0.028	VWB-SWB: 0.334 (0.08–1.74)
	Both victims and witnesses of bullying/mobbing	53	2.25	1.072	0.147	1.95	2.54			
								0.026		
3. It seemed as if I was reliving the bullying/mobbing experience I had.	Solely bullying/mobbing victims	24	1.96	1.160	.237	1.47	2.45			
	Solely witnesses of bullying/mobbing to others	12	1.25	0.452	0.131	0.96	1.54		0.058	VWB-SWB: 0.348 (−0.02–1.74)
	Both victims and witnesses of bullying/mobbing	53	2.09	1.148	0.158	1.78	2.41			
								0.058		
4. I had trouble sleeping after the bullying/mobbing experience I had.	Solely bullying/mobbing victims	24	1.79	1.179	0.241	1.29	2.29			
	Solely witnesses of bullying/mobbing to others	12	1.08	.289	0.083	.90	1.27			
	Both victims and witnesses of bullying/mobbing	53	1.72	1.007	0.138	1.44	1.99			
								0.107		
5.I felt discouraged about the future after the bullying/mobbing experience I had.	Solely bullying/mobbing victims	24	2.00	1.216	0.248	1.49	2.51			
	Solely witnesses of bullying/mobbing to others	12	1.17	.577	0.167	.80	1.53		0.030	VWB-SWB: 0.391 (0.08–2.03)
	Both victims and witnesses of bullying/mobbing	53	2.23	1.325	0.182	1.86	2.59			
								0.030		
6. Reminders of the bullying/mobbing experience I had upset me.	Solely bullying/mobbing victims	24	1.92	0.929	0.190	1.52	2.31			
	Solely witnesses of bullying/mobbing to others	12	1.50	0.905	0.261	.93	2.07			
	Both victims and witnesses of bullying/mobbing	53	2.08	1.071	0.147	1.78	2.37			
								0.208		
7. I had little interest in being around others after the bullying/mobbing experience I had.	Solely bullying/mobbing victims	24	2.00	1.022	0.209	1.57	2.43			
	Solely witnesses of bullying/mobbing to others	12	1.42	0.669	0.193	.99	1.84			
	Both victims and witnesses of bullying/mobbing	53	1.83	1.105	0.152	1.53	2.13			
								0.285		
8. I felt jumpy after the bullying/mobbing experience I had.	Solely bullying/mobbing victims	24	2.13	1.076	0.220	1.67	2.58			
	Solely witnesses of bullying/mobbing to others	12	1.33	.778	0.225	0.84	1.83		0.048	VWB-SWB: .371 (0.01–1.85)
	Both victims and witnesses of bullying/mobbing	53	2.26	1.258	0.173	1.92	2.61			
								0.047		
9. I was less active than usual after the bullying/mobbing experience I had.	Solely bullying/mobbing victims	24	2.13	1.329	0.271	1.56	2.69			
	Solely witnesses of bullying/mobbing to others	12	1.58	0.793	0.229	1.08	2.09	0.152		
	Both victims and witnesses of bullying/mobbing	53	2.13	1.359	0.187	1.76	2.51			
								0.399		
10. I thought about the bullying/mobbing experience I had when I didn’t intend to.	Solely bullying/mobbing victims	24	2.13	1.361	0.278	1.55	2.70			
	Solely witnesses of bullying/mobbing to others	12	1.50	0.674	0.195	1.07	1.93			
	Both victims and witnesses of bullying/mobbing	53	2.00	1.092	0.150	1.70	2.30			
								0.282		
11. I had trouble concentrating after the bullying/mobbing experience I had.	Solely bullying/mobbing victims	24	1.83	1.090	0.223	1.37	2.29			
	Solely witnesses of bullying/mobbing to others	12	1.25	0.452	0.131	0.96	1.54			
	Both victims and witnesses of bullying/mobbing	53	1.85	0.969	0.133	1.58	2.12			
								0.140		
12. I avoided people, places, or things that reminded me of the bullying/mobbing experience I had.	Solely bullying/mobbing victims	24	1.96	1.268	0.259	1.42	2.49			
	Solely witnesses of bullying/mobbing to others	12	1.50	1.000	0.289	0.86	2.14			
	Both victims and witnesses of bullying/mobbing	53	2.26	1.318	0.181	1.90	2.63			
								0.152		
13. I had disturbing dreams about the bullying/mobbing experience I had.	Solely bullying/mobbing victims	24	1.58	1.213	0.248	1.07	2.10			
	Solely witnesses of bullying/mobbing to others	12	1.08	0.289	0.083	0.90	1.27			
	Both victims and witnesses of bullying/mobbing	53	1.53	0.912	0.125	1.28	1.78			
								0.292		
14. I wanted to avoid working with some people linked with the bullying/mobbing experience I had.	Solely bullying/mobbing victims	24	2.25	1.359	0.277	1.68	2.82			
	Solely witnesses of bullying/mobbing to others	12	1.42	0.793	0.229	0.91	1.92		0.006	VWB-SWB: 0.391 (0.31–2.26)
	Both victims and witnesses of bullying/mobbing	53	2.70	1.234	0.169	2.36	3.04			
								0.005		
15. I was easily annoyed after the bullying/mobbing experience I had.	Solely bullying/mobbing victims	24	1.83	0.917	0.187	1.45	2.22		0.027	VWB-SVB: 0.288 (0.07–1.51)
	Solely witnesses of bullying/mobbing to others	12	1.75	1.138	0.329	1.03	2.47			
	Both victims and witnesses of bullying/mobbing	53	2.62	1.274	0.175	2.27	2.97			
								0.007		
16. I expected something bad to happen after the bullying/mobbing experience I had.	Solely bullying/mobbing victims	24	1.58	0.881	0.180	1.21	1.96			
	Solely witnesses of bullying/mobbing to others	12	1.50	0.798	0.230	0.99	2.01			
	Both victims and witnesses of bullying/mobbing	53	2.02	1.101	0.151	1.72	2.32			
								0.108		
17. I noticed gaps in my memory about the bullying/mobbing experience I had.	Solely bullying/mobbing victims	24	1.54	0.833	0.170	1.19	1.89			
	Solely witnesses of bullying/mobbing to others	12	1.17	0.389	0.112	0.92	1.41			
	Both victims and witnesses of bullying/mobbing	53	1.83	1.156	0.159	1.51	2.15			
								0.100		
Total score of STSS-M	Solely bullying/mobbing victims	24	33.21	14.903	3.042	26.92	39.50			
	Solely witnesses of bullying/mobbing to others	12	23.75	7.436	2.147	19.03	28.47		0.019	VWB-SWB: 4.23 (1.69–22.81)
	Both victims and witnesses of bullying/mobbing	53	36.00	13.446	1.847	32.29	39.71			
								0.018		

*Mean and standard deviation, as well as 95% Confidence Intervals for the total STSS-M score and individual STSS-M items per SWB, VWB, and SVB subgroup are presented.*

With regard to the socio-demographic, educational and employment characteristics of the sample, the only statistically significant difference was found in relation to the work department (ANOVA, *F* = 3.688, *p* = 0.029). Specifically, those employed in EDs reported a higher mean STSS-M total score [M(SD): 39.88(15.12)] compared to those employed in ICUs [M(SD): 31.07(12.29)] [*p* = 0.03, 95% CI: 3.26 (0.68–16.93)]. Also, a statistically significant positive mild association was noted between the STSS-M total score and emotional the exhaustion score (*r* = 0.273, *p* = 0.01).

Table 3 presents mean differences in the intensity of workplace bullying-related trauma symptoms as assessed by the STSS-M total score in relation to the variables included in the WVHS-CCSRI Part C.II-M questionnaire. Specifically, those considering to resign reported more intense trauma symptoms compared to those that had never thought about resigning due to bullying/mobbing experiences (*p* = 0.008). Regarding the reported frequency of bullying/mobbing in the workplace, those who reported a high frequency of the phenomenon, i.e. “quite to very often,” reported more intense trauma symptoms compared to those who declared a moderate frequency (*p* = 0.018). Finally, the participants who had been punished for reporting a bullying/mobbing incident stated more intense trauma symptoms compared to those who did not (*p* = 0.001), as did those who considered the incident unimportant to be reported compared to those who avoided reporting due to other causes (*p* = 0.048).

**TABLE 3:** Mean differences in the degree of workplace bullying-related trauma symptoms according to STSS-M total score between different variable groups of the Part C.II-M of the WVHS-CCSRI.

		95% Confidence Interval for Mean								
Workplace bullying/mobbing relevant variable	Variable subgroup	*N*	STSS-M Mean	Std. Deviation	Std. Error	Lower bound	Upper bound	*p* value (ANOVA, *t*-test)	*Post-hoc* analysis (Scheffe test)	
								R2C5.34		Subgroup Difference: Std. Error (95% CI)

Have you ever resigned or considered resigning because of bullying/mobbing experiences in your workplace?	Yes, I did (A)	3	43.67	7.371	4.256	25.36	61.98			
	I thought of but did not (B)	18	43.44	13.815	3.256	36.57	50.31		0.008	B-C: 3.539 (2.60–20.29)
	No (C)	56	32.00	12.985	1.735	28.52	35.48			
								0.004		
How often, to your opinion, bullying/mobbing happens in your workplace?”	Quite to very often (A)	28	39.68	14.368	2.715	34.11	45.25		0.018	A-B: 3.418 (1.44–18.53)
	Moderately (B)	21	31.95	12.863	2.807	26.10	37.81			
	Not at all/rarely (C)	26	29.69	9.915	1.944	25.69	33.70			
								0.012		
Have you ever been punished because you reported a workplace bullying/mobbing incident?	No	83	32.31	13.001	1.427					
	Yes	6	51.33	11.894	4.856				0.001	5.470 (–29.893–8.14)
If you did not report or tell about the bullying/mobbing to others, you did so because you thought it was unimportant.	Yes	54	37.17	13.855	1.885					
	No	23	30.35	12.995	2.710				0.048	3.388 (0.069–13.569)

In multivariable forward stepwise logistic regression analysis, in which age between 26 and 35 years, work experience less than 5 years, single family status, Master’s degree education, ED work setting, emotional exhaustion, reporting of “quite to very often” frequency of workplace bullying/mobbing occurrence, being punished for reporting a bullying/mobbing incident, and considering bullying/mobbing experience as unimportant to be reported were included as predictors of workplace bullying-related trauma symptoms, none of them retained a statistically significant association with the dependent variable (beta = −249.0; *B* = 0.000, *p* = 1.00) (see Supplementary Material for relevant data).

## Discussion

The findings of the present study confirmed the main hypothesis on the association of workplace bullying-related trauma symptoms with (a) emotional exhaustion, a work-related stress indicator, and (b) employment in EDs, an occupational factor. This data was confirmed in a sample of Greek-Cypriot nurses working in emergency and critical care settings. Yet, we did not confirm any other association between workplace bullying-related trauma symptoms and educational or any other employment and demographic variables. Additionally, the present data did not confirm any predictors of workplace bullying-related trauma symptoms. Instead, we confirmed our hypothesis on the association between workplace bullying/mobbing relevant experiences and a) socio-demographic (26–35 years of age, single marital status), educational (Master’s education) and occupational (less than 5 years work experience, district of employment) variables, and b) emotional exhaustion.

Nonetheless, to the best to our knowledge, this is the first study exploring the link between workplace bullying/mobbing and associated traumatic symptoms in nurses employed in private and public emergency and critical care settings in Cyprus. Although previous data confirms increased rates of workplace bullying/mobbing among nurses, only few have addressed critical care and emergency nurses, internationally, and even fewer have looked into relevant traumatic symptoms (Yun et al., 2014; Nielsen et al., 2015; Chatziioannidis et al., 2018; Bambi et al., 2018). Specifically, the present data revealed that approximately four out of five participants (78.8%) reported a workplace bullying/mobbing experience as a victim, a witness or both, while approximately two out of three (68.1%) reported bullying/mobbing victimization. Also, one out of three of those who reported bullying/mobbing experiences (35.9%) reported moderate to high intensity of relevant trauma symptoms. Previous studies in nurses report increased rates of workplace bullying/mobbing victimization, even up to 81% (Spector et al., 2014; Difazio et al., 2019), a percentage similar to the one reported herein. Thus, our findings confirm previous data reporting increased frequency of workplace bullying/mobbing among nurses. Specifically, it has been previously shown that approximately one out of four staff nurses may be a victim of workplace bullying/mobbing (Wilson, 2016). Regarding critical and emergency nurses, data from Israel shows that approximately one out of three ICU nurses may be a workplace bullying/mobbing victim (Ganz et al., 2015), a percentage lower than the one reported herein (i.e. 61.6%), while a study in Jordanian emergency nurses showed that 90% of the participants reported relevant experiences (Al-Ghabeesh and Qattom, 2019), a frequency much higher than the one stated in the present study (i.e. 29.3%).

Most importantly, the present data points out not only the extent of these phenomena, but also the link between bullying/mobbing experience and relevant trauma symptoms. Indeed, although previous studies confirmed increased manifestation of PTSD symptoms in critical care nurses (Karanikola et al., 2015), the present study associated these symptoms with bullying/mobbing phenomena. Furthermore, those who reported both victimization and witnessing of workplace bullying/mobbing to others (VWB) reported more intense traumatic symptoms compared to those who only witnessed these phenomena (SWB) (see Table 2). This difference denotes that although both experiences (i.e. witnessing of bullying to others/bullying victimization) may be traumatic, being simultaneously a victim and a witness of bullying/mobbing seems to have a more severe impact on one’s mental health. This may also reflect that prolonged or repeated exposure to workplace bullying/mobbing via multiple forms may have a more intense impact on one’s psychological and mental status.

Another important finding herein was that those who reported both victimization and witnessing of workplace bullying/mobbing to others reported a higher degree of emotional exhaustion compared to those with no workplace bullying/mobbing experiences, which supports the association between bullying/mobbing victimization and burnout indices. This is in line with previous evidence showing a relation between exposure to workplace bullying/mobbing and burnout symptoms; indeed, workplace bullying has been identified as an important predictor of burnout and work-related emotional exhaustion in nurses (Allen et al., 2015; Ajoudani et al., 2019). Emotional exhaustion is a feature associated with prolonged exposure to a stressful work environment, as well as with an intention to quit the job (Schadenhofer et al., 2018). Interestingly, previous data shows that although critical care nurses are supposed to have a more demanding clinical role than emergency nurses, yet the latter seem to be almost three times more likely to experience chronic fatigue, work-related stress and burnout, along with exposure to incivility the workplace (Abdul Rahman et al., 2017). Overall, a number of job-related problems have been linked with bullying/mobbing exposure in nurses (Olsen et al., 2017; Finstad et al., 2019), while there is considerable evidence on the positive association between bullying/mobbing victimization and burnout in them (Waschgler et al., 2013; Allen et al., 2015). However, due to the cross-sectional design of the present study, it is not clear: (a) if bullying/mobbing experiences may be deemed as a trigger of emotional exhaustion; (b) if increased work-related stress in emergency and critical care settings is a cause of bullying/mobbing incidents; and (c) even if those experiencing higher levels of work-related stress are more vulnerable to bullying/mobbing victimization. Further longitudinal studies to clarify these issues in this population are needed. Nevertheless, measures to reduce workplace bullying/mobbing are expected to alleviate nurses from burnout, as well.

Although a higher frequency of bullying/mobbing occurrence was described in ICU compared to ED and CCCU participants, the intensity of traumatic symptoms was more severe in ED participants compared to ICU participants. This is in accordance with the finding that the participants employed in EDs thought of quitting the job because of workplace bullying/mobbing experiences more frequently compared to participants employed in other settings (CCCUs and ICUs) (see Table 3). A possible explanation for this may lie within the different copying strategies and degree of resilience in the two populations (Mealer et al., 2017), or in the differences in the organizational culture and supportive procedures in different work settings (Sheehan et al., 2018; Sungur et al., 2019). In any case, only a very low percentage of the participants herein considered quitting the job due to bullying/mobbing experiences (18/77; see Table 3), while even fewer did resign due to these phenomena (3/77; see Table 3). This is in line with previous data in other European countries showing that almost only one out of nurses consider quitting their job due to bullying/mobbing experiences (Bambi et al., 2017).

Although the present study confirmed a previously identified association between exposure to workplace bullying/mobbing and traumatic symptoms (Nielsen et al., 2015), we observed a higher prevalence of self-reported traumatic symptoms after exposure to workplace bullying/mobbing (i.e. 35.9%) compared to other studies. A recent literature review identified that around 10% of the nurses who reported exposure to workplace bullying/mobbing may develop PTSD symptoms (Bambi et al., 2018). The discrepancy between this data and the present results may be attributed to a number of reasons, including differences in the tools for PTSD symptoms assessment or different cut-off points used for data interpretation. Yet, it should be stressed that neither STSS-M nor other similar instruments are diagnostic of PTSD; instead, the focus of relevant instruments is on identifying differences of the experienced PTSD symptoms across time or need for advanced assessment and support. Although the present data identified that a significant percentage of nurses experience debilitating psychological traumas after exposure to workplace bullying/mobbing, it is worth noting that the majority of them still show a tendency towards symptom underestimation and non-treatment seeking behaviors (Aristidou et al., 2019). Moreover, in the present study a vast majority of participants (54/77; see Table 3) responded that they avoided reporting the bullying/mobbing experience because of thinking it was not important. Nonetheless, the finding that those not reporting the experiences had elevated trauma symptoms is intriguing, and suggests that organizational policies must include interventions to enhance the nurses’ ability to recognize bullying/mobbing behaviors and their impact (Obeidat et al., 2018). Policies should also empower those with relevant experiences not only to report incidents of bullying/mobbing, but most importantly support them to effectively manage trauma-related symptoms. Special focus is expected to be given on those who have been shown to be more frequently exposed to bullying/mobbing phenomena, i.e. nurses aged 26–35 years, single, with Master’s education, and with less than 5 years of work experience. In addition, the present study revealed differences in the workplace bullying/mobbing occurrence and in trauma symptoms frequency in relation to the different work settings. These differences may be attributed to the different response rates between different ICUs, or between ICUs and EDs, as well as to differences in relation to workload and subsequent work-related stress, organizational culture, quality of professional relations and support among peers (An and Kang, 2016).

Given that the majority of workplace bullying/mobbing victims do not seek support, assessment for both PTSD symptoms and exposure to workplace bullying/mobbing must take place regularly to support early detection, treatment and intervention. At the same time, since the participants who reported more intense traumatic symptoms had more frequently considered quitting the job, implementation of relevant preventive measures is necessary to retain nurses into the profession. Most importantly, measures to support and empower those exposed to bullying/mobbing incidents are much needed, with special focus on the organizational parameters that may trigger increased work-related stress and subsequent bullying/mobbing phenomena.

Moreover, the present results revealed an association between the intensity of traumatic symptoms and the frequency of workplace bullying, showing that the higher the frequency of workplace bulling/mobbing occurrence the more intense the experience of traumatic symptoms. Data from the general nursing population is in line with this finding, which underline that those who frequently report daily workplace bullying/mobbing exposure are also showing significantly higher levels of stress, anxiety and PTSD symptoms, compared to those who state infrequent exposure to workplace bullying/mobbing behaviors (Berry et al., 2016).

It was also shown herein that the overall mean satisfaction from work was lower than the mean satisfaction from any other work-related domain assessed. Further research into the work conditions and professional satisfaction in emergency and critical care nurses in Cyprus may provide a robust explanation for these results.

### Limitations

The present findings need to be viewed in light of a several limitations. The most important limitation is the low response rate (RR) (32,56%). Indeed, the few existing studies in ED and ICU nurses have produced higher response rates. This includes both international studies (e.g. Al-Ghabeesh and Qattom, 2019, in Jordan; response rate 89.6% and Ganz et al., 2015, in Israel; response rate not reported; 156 ICU nurses participated from five medical centers), and studies performed in nurses in Cyprus. The response rate in Cypriot ED and ICU nurses was 63.3% in the study by Rousou and Pavlakis (2011), 76.7% in the study by Raftopoulos and Pavlakis (2013), 82% in the study by Lambrou et al. (2015), and 92.8% in the study by Evripidou et al. (2015). It is of note that the demographics of nurses in these studies were quite similar to the present one in terms of male/female ratio, age, educational level and years of experience. This data allows us to suggest that despite the low response rate in the present study, our findings may be partially generalizable to the emergency and critical care nursing population in Cyprus. That said, we do not have an adequate explanation for the relatively low response rate of the nurses in our study. This because the researchers tried to explain as much as possible the process and the importance of the study, as well as issues of confidentiality and anonymity, aiming to achieve as high response rate, as possible. However, one possible explanation for this may involve increased workload or reduced staffing in each shift during the data collection period. Specifically, the period from October to December is considered in Cyprus with a high rate of sick leaves due to seasonal infections. Nevertheless, the reluctance of nurses to respond to anonymous questionnaires about their work conditions is distressing, likely requiring necessary interventions in the organizational climate of these units; as nurses unwilling to participate in such studies may be more likely to have been themselves victims of workplace bullying/mobbing.

Another limitation of the study was the small number of participants who only witnessed bullying/mobbing to others. Even so, the present finding can inform future investigations. An additional limitation may be the lack of triangulation of data via colleagues/nomination techniques, since only self-reported data was collected. This may have resulted in an overestimation or underestimation of the frequency of bullying/mobbing experiences. Nonetheless, data coming from other countries confirms the frequency of workplace bullying/mobbing presented herein (Bambi et al., 2019).

## Conclusion

The present data highlights the need to establish effective procedures for bullying/mobbing reporting, that will support bulling/mobbing victims and witnesses, and to further protect their legal rights. Documentation and management of incidents of incivility in the workplace is expected to improve the perception of justice and safety in the workplace.

What this study also proposes is that both victims and witnesses of workplace bullying/mobbing need to be assessed by mental health professionals for PTSD symptoms in order to have access to effective treatment. Overall, the present findings support the need improvement of current organizational culture, and for revision of the policy against bullying, violence and harassment in public emergency and critical care settings in Cyprus. Changing the culture regarding incivility and the attitudes toward the victims of bullying/mobbing is crucial.

## Data Availability Statement

The raw data supporting the conclusions of this article will be made available by the authors.

## Ethics Statement

This study was reviewed and approved by the Cyprus National Bioethics Committee. The participants provided their written informed consent to participate in this study.

## Author Contributions

LA participated in study design, data collection and data analysis, and drafted the manuscript. MM participated in data interpretation and in writing part of the manuscript. EP participated in data interpretation and critical review of the final manuscript. NM participated in data analysis and interpretation of the results. MK participated in study design, data analysis, interpretation of data, and in writing part of the manuscript. MK and MM were the supervisors of LA, from which this manuscript was produced, having contributed equally to the study with LA. All of the authors have read and approved the final manuscript.

## Conflict of Interest

The authors declare that the research was conducted in the absence of any commercial or financial relationships that could be construed as a potential conflict of interest.
