# Supplementary material for: Association Between Workplace Bullying Occurrence and Trauma Symptoms Among Healthcare Professionals in Cyprus
Source: Front Psychol. 2020 Nov 12;11:575623. doi: 10.3389/fpsyg.2020.575623 (PMC7688662; doi:10.3389/fpsyg.2020.575623)
Supplement: Supplementary file 1 [file Data_Sheet_1.doc]

**Bullying/ mobbing questionnaire**

| Below are some questions about bullying/mobbing in the workplace. Bullying/mobbing in the workplace is about recurring and over time malicious and offensive physical, verbal or interpersonal behaviours aiming to humiliate and undermine an employee or a group of employees by a person or group of persons. Workplace bullying/mobbing takes place through threatening, offensive, derogatory, dismissive behaviours, as well as acts that cause terror and intense mental pressure on the receivers. Please read these questions carefully and note √ on the box with the answer that represents you. It is quite important to answer all questions honestly. | | | | | | |
| --- | --- | --- | --- | --- | --- | --- |
| **1.** | **In the last 12 months, have you been bullied / mobbed in your workplace?** | Yes | No |  |  |  |
| **2.** | **In the last 12 months, have you witnessed bullying/ mobbing towards other people in your workplace?** | Yes | No |  |  |  |
| **3.** | **Ηow often have you been bullied / mobbed or witnessed bullying/mobbing towards others in your workplace during the last 12 months,?** | Almost every day | 4-5 times/year | 4-5 times/ month | 1-2 times in total |  |
| **4.** | **Please think of the last time you were bullied mobbed (or witnessed bullying/mobbing towards others) in your workplace. Who bullied/ mobbed you/ was the bully?** | Administrative personnel | Staff nurse | External colleague/worker | Patient/ Client | Relatives of patient/ client |
| **5.** | **The last time you were bullied/mobbed, the person who intimidated you was of:** | Equal ranking with you | Superior ranking with you | Inferior ranking with you | Had no ranking relation with you |  |
| **6.** | **Where did the bullying / mobbing incident take place?** | Inside ICU/ED/CCCU | Outside IC/ED/CCCU | Other (please, clarify)……………………………………….  ……………………………………………………………………… | | |
| **7.** | **How was the bullying/ mobbing expressed?** | Verbally | Physical violence/ gestures | Gossip/ rumours | Isolation/ Information hiding | Irrelevant duties assignments/ unrealistic deadlines assignment  **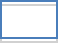** |
| **8.** | **Do you think the bullying/ mobbing incident could have been prevented?** | Yes | No | If yes, please, please describe in short the way……………………………………….  ……………………………………………………………………… | | |
| **9.** | **Was any action taken**  **to investigate the causes of the bullying/mobbing?** | Yes | No | I don’t know | If yes, by whom?  ………………………………………….  ………………………………………… | |
| **10.** | **Please think of the last time you were bullied/ mobbed in your workplace. How satisfied are you with the manner in which the incident was handled?** | Very dissatisfied | Dissatisfied | Νeither satisfied nor dissatisfied | Satisfied | Very satisfied |
| **11.** | **Please think of the last time you were bullied/ mobbed in your workplace. What were the consequences for the person who bullied/ mobbed you?** | None | Reported to police | Verbal warning issued | Aggressor prosecuted | Job discontinued |
| **12.** | **Please think of the last time you were bullied/ mobbed in your workplace. What kind of support did your employer or supervisor offer to provide you with?** | No support at all | Counselling | Opportunity to speak about/report it | Other support. (please, clarify)………………………………  ………………………………………….. | |
| **13.** | **How often, to your opinion, bullying/ mobbing happens in your workplace?** | Rarely/never | Not very frequently | Moderately frequently | Very frequently | Highly frequently |
| **14.** | **Have you ever been punished because you reported a workplace bullying/ mobbing incident?** | Yes | No |  |  |  |

| **15. How did you respond to the most recent incident bullying / mobbing in your workplace? Please tick all relevant boxes** | | | |
| --- | --- | --- | --- |
| Took no action |  | Told the bully to stop |  |
| Told a colleague |  | Sought counselling |  |
| Sought help from the Union/ Association |  | Tried to defend myself physically |  |
| Τransferred to another position |  | Pursued prosecution |  |
| Tried to pretend it never happened |  | Told friends/family |  |
| Reported the incident to a senior staff member |  | Asked support from the anti-bullying committee of the hospital |  |
| Filled-in the incident/ complaint form |  | Filled-in a compensation claim |  |

**16. How satisfied are you with the way you handled the most recent bullying/ mobbing behavior you have experienced in your workplace?**

|  |  |  |  |  |  |  |  |  |  |
| --- | --- | --- | --- | --- | --- | --- | --- | --- | --- |

**0 1 2 3 4 5 6 7 8 9 10**

| **17. If you did not report or tell about the incident of workplace bullying to others, why not? Please tick all relevant boxes** | | | |
| --- | --- | --- | --- |
| I thought that the incident was not important |  | Felt ashamed |  |
| Felt guilty |  | Afraid of negative consequences |  |
| Did not know who to report it to |  | Thought that there was nothing to be done to eliminate this behavior (useless) |  |
| Anti-bullying policy is not provided by the institution |  | Colleagues and peers asked/advised me not to report/ take actions against |  |
| Other: | | |  |

| **18. How did you respond to the most recent incident of workplace bullying / mobbing that you witnessed? Please tick all relevant boxes** | | | |
| --- | --- | --- | --- |
| I advised the bullying victim to kindly ask the bully to stop this behavior |  | I advised the bullying victim not to take action until the incidence was repeated |  |
| I myself reported the workplace incident to the manager |  | I took no action |  |
| I myself asked the bully to stop |  |  |  |
| Other (please explain) | | | |

**19. Have you ever resigned or considered resigning because of bullying/mobbing experiences in your workplace?**

| **Yes, I did.** |  |
| --- | --- |
| **I thought of but did not.** |  |
| **No, I did not.** |  |
