# Supplementary material for: Association Between Workplace Bullying Occurrence and Trauma Symptoms Among Healthcare Professionals in Cyprus
Source: Front Psychol. 2020 Nov 12;11:575623. doi: 10.3389/fpsyg.2020.575623 (PMC7688662; doi:10.3389/fpsyg.2020.575623)
Supplement: Supplementary file 2 [file Table_1.docx]

| **Supplementary Table**. Adjusted odds ratios (and 95% CI) of workplace bullying-related trauma symptoms by socio-demographic, employment and bullying/mobbing-related attitudes as estimated in multivariable forward stepwise logistic regression analysis. | | | | | |
| --- | --- | --- | --- | --- | --- |
| **Socio-demographic, employment and bullying/mobbing-related attitudes** | B | S.E  . | Wald | Df | p values |
| **Age**  Other age group participants  **26-35 years** | 19.5 | 38098.7 | .000 | 1 | 1.00 |
| **Family status**  Married  **Single** | -8.7 | 12975.8 | .000 | 1 | 0.99 |
| **Education**  Only BSc degree  **Master’s degree** | 2.6 | 14066.4 | .000 | 1 | 1.00 |
| **Working experience**  Other  **Less than 5 years** | 106.3 | 40523.7 | .000 | 1 | 1.00 |
| **Working setting**  ICU  **ED** | -1.184 | 14064.0 | .000 | 1 | 1.00 |
| **Emotional exhaustion** No  **Yes** | 3.002 | 2980.4 | .000 | 1 | .999 |
| **How often, to your opinion, bullying/ mobbing happens in your workplace?**  Moderately/Not at all/Rarely Other  **Quite to very often** | -20.831 | 16027.0 | .000 | 1 | .999 |
| **Have you ever been punished because you reported a workplace bullying/ mobbing incident?**  No  **Yes** | 27.715 | 9295.9 | .000 | 1 | .998 |
| **If you did not report or tell about the bullying/mobbing to others, you did so because you thought it was unimportant**  No  **Yes** | 3.974 | 18940.9 | .000 | 1 | 1.00 |
| **Constant** | -249.0 | 138918.9 | .000 | 1 | 1.00 |
|  |  |  |  |  |  |
